# Evaluation of [^3^H]SynVesT‐1 binding parameters in post‐mortem brain tissue from control and Alzheimer's Disease

**DOI:** 10.1002/alz70856_103316

**Published:** 2025-12-26

**Authors:** Laetitia Lemoine, Laura Kulagowska, David Bonsall, Declan King, Scott Gladstein, Amanda Bevis, Martin Sabandal, Lisa Wells, Sjoerd J Finnema, Tara L. Spires‐Jones, FNIH SV2A Project Team

**Affiliations:** ^1^ Perceptive, London, United Kingdom; ^2^ Centre for Discovery Brain Sciences at the University of Edinburgh, Edinburgh, Scotland, United Kingdom; ^3^ UK Dementia Research Institute at the University of Edinburgh, Edinburgh, Scotland, United Kingdom; ^4^ Abbvie Inc., North Chicago, IL, USA; ^5^ Foundation for the National Institutes of Health, North Bethesda, MD, USA

## Abstract

**Background:**

There is currently a need for an imaging biomarker of synaptic density that would allow the tracking of changes associated with neurodegeneration, disease progression and possible therapeutic response. The aim of this study was to use homogenate binding techniques to assess [^3^H]SynVesT‐1 binding to synaptic vesicle glycoprotein 2A (SV2A), found in pre‐synaptic terminals, to understand its potential as a synaptic density biomarker.

**Method:**

Postmortem brain tissues from 70 subjects, across 3 different regions (entorhinal cortex (EC), cerebellum and centrum semiovale) and two homogenate fractions (total homogenate (TH) and synaptoneurosome (SN)) were used to determine the binding parameters (binding affinity (K_D_), and maximal binding sites (B_MAX_)) of [^3^H]SynVesT‐1. Sex‐matched subjects were initially divided into 3 groups: control (Braak Stage 0‐II), early Alzheimer's Disease (E‐AD) (Braak Stage III‐IV) and late AD (L‐AD) (Braak Stage V‐VI).

**Result:**

A saturable specific binding signal was observed in EC and cerebellum homogenates from all three groups. A suitable binding curve could not be applied to the centrum semiovale, with less specific binding, and greater variability across cases. The K_D_ of [^3^H]SynVesT‐1 was comparable across groups and homogenate preparations (TH and SN), ranging from 3‐7 nM. In initial groupwise analyses, the B_MAX_ ranged from 2278 ± 101 fmol/mg protein in L‐AD, to 2788 ± 109 fmol/mg protein in control in the TH preparations from the EC. SN homogenates for the EC show B_MAX_ ranged from 3958 ± 183 fmol/mg protein in L‐AD, to 5193 ± 316 fmol/mg protein in E‐AD. In cerebellum TH, B_MAX_ ranged from 2263 ± 88 fmol/mg protein in control, to 2681 ± 186 fmol/mg protein in E‐AD. In cerebellum SN homogenates, B_MAX_ ranged from 2790± 134 fmol/mg protein in L‐AD, to 4085 ± 239 fmol/mg protein in E‐AD.

**Conclusion:**

Initial results show group‐wise differences in the B_MAX_ of [^3^H]SynVesT‐1 in EC, with E‐AD subjects showing greater binding signal. This may suggest compensatory mechanisms occurring early in the progression of the disease. This is part of the SV2A PET Project, a program of the FNIH Biomarker Consortium.

